# Supplementary material for: Potential-dependent transition of reaction mechanisms for oxygen evolution on layered double hydroxides
Source: Nat Commun. 2023 Jul 15;14:4228. doi: 10.1038/s41467-023-40011-8 (PMC10349880; doi:10.1038/s41467-023-40011-8)
Supplement: Supplementary file 1 — Supplementary Information [file 41467_2023_40011_MOESM1_ESM.pdf]

Supplementary Information for

Potential-Dependent Transition of Reaction Mechanisms for Oxygen Evolution on Layered Double Hydroxides

Zeyu Wang[1], William A. Goddard III[2], Hai Xiao[1]*

[1]Department of Chemistry and Key Laboratory of Organic Optoelectronics and Molecular Engineering of Ministry of Education, Tsinghua University, Beijing 100084, China

[2]Materials and Process Simulation Center, California Institute of Technology, Pasadena, CA 91125, USA

*Corresponding author. E-mail: haixiao@tsinghua.edu.cn

Additional Computational Details

1. Structural models

The active phase of Ni-based LDHs for OER has been identified as the γ-phase, in which the intercalated cations such as K^+ modulate the average oxidation state (OS) of Ni(M) to be +3.6~3.7. [1-3] However, the γ-phase is of low crystallinity and high structural complexity due to the intercalated species, while its precursor, the β-phase, is well-defined. Thus, we build the models of γ-phase based on the β-phase by removing 3/4 of interlaminar protons to render an average OS of +3.75 for Ni(M) [4] and adopting the active (100) edges. [5, 6] For Ni(M)OOH (M = Fe, Co), 1/4 of Ni are substituted by M to match the ratio in the best-performing catalysts. [7, 8] For the DFT calculations of OER on Ni(M)OOH, a (2×1) supercell slab of (100) edge containing 4 layers (each layer with 4 (Ni, M)O_2 units) was used to avoid the possible adsorbate interactions between two neighboring cells, with the reciprocal space sampled by a grid of 3 × 7 × 1. Periodic boundary conditions were applied in all directions with a vacuum space of at least 15 Å in the direction perpendicular to the edge in order to eliminate spurious image-image interactions. The optimized lattice constants of Ni(M)OOH are listed in Table S1. We note that the OER activity may be influenced by the spacing between layers in the LDHs, so we calculate the reaction free energies of electrochemical steps in AEM on NiOOH with varying its layer spacing (Figure S5), and find insignificant changes in the reaction free energies. Therefore, we believe that our models serve as suitable representations of the working catalysts in experiments.

All structures were fully optimized (with the bottom two layers fixed, shown in Figure S6-8) with convergence thresholds of 10^{-5} eV for SCF and 0.03 eVÅ^{-1} for the maximal force, and tighter convergence thresholds of 10^{-6} eV for SCF and 0.02 eVÅ^{-1} for the maximal force were used for the transition state search and vibrational frequency calculations.

The under-coordinated TM and bridge O sites on the edge may be saturated by OH and H, respectively, via the dissociative adsorption of H_2O from electrolyte. However, our constant potential calculations (Figure S4) suggest a dehydration of the surface where the under-coordinated TM and bridge O sites are exposed. The optimized structures of all states for OER on Ni(M)OOH are shown in Figures S2-4.

We also performed the magnetic moment analysis of the NiOOH matrix to confirm the average OS of +3.75 for Ni (Figure S11), which indicates mixed Ni^{3+} and Ni^{4+} at the OER-working potential to render the average OS of +3.75. In addition, we calculated the grand-canonical free energy changes for the electrochemical deprotonation (oxidation) of all the surface models (Figure S12), and the results indicate that it becomes thermodynamically favorable (exothermic) to deprotonate (oxidize) the surfaces to reach the average OS of +3.75 at the working potentials for OER on each surface. Note that all the structures are ferromagnetic and remain so after the adsorptions of intermediates under different potentials. Table S5 lists the total magnetic moments for all states at different potentials, and Figures S13 show the atomic spin densities of catalytically active metal sites in the states before O-O coupling.

2. Computational hydrogen electrode (CHE) method

The CHE method was employed for calculating the Gibbs free energy of proton-electron pair as[9]

$$G(H^+) + G(e^-) = 1/2\ G(H_2) - eU$$

where $G(H^+)$ and $G(e^-)$ are the Gibbs free energies of proton and electron, respectively; $G(H_2)$ is the Gibbs free energy of H_2 at standard conditions; U is the applied potential referenced to the reversible hydrogen electrode (RHE). We assumed here an alkaline environment with pH = 14 for the OER operating electrode, but the pH contribution is cancelled because of the RHE scale used for U.

Correspondingly, the reaction free energy of OER ($2H_2O(l) \rightarrow O_2\ (g) + 4H^+\ (aq) + 4e^-$) is expressed as

$$\Delta G = 4 \times \left(\frac{1}{2} G_{H_2}\right) + G_{O_2} - 2G_{H_2O(l)}$$

and the experimental value of ΔG is 4.92 eV at the RHE scale. G_{H_2} was calculated as an isolated gas molecule (in a large unit box of 12×12×15 Å^3) with the free energy contributions from translation, rotation, and vibration assuming the ideal-gas approximation. $G_{H_2O(l)}$ was calculated in the same way as G_{H_2} but corrected with a $k_B T \ln p/p°$ term that corresponds to the saturation vapor pressure ($p/p° = 0.031$) in equilibrium with liquid water at room temperature. Finally, G_{O_2} was derived from the expression of $\Delta G = 4.92$ eV. All values of G are listed in Table S2.

3. Free energy correction

The free energy of each state is defined as

$$G = E_{\mathrm{DFT}} + E_{\mathrm{ZPE}} + H - TS$$

where E_{DFT} is the DFT-calculated electronic energy; E_{ZPE}, H, and TS represent zero-point energy, enthalpy, and entropy obtained by the vibrational frequency calculations. Note that we set a threshold for low frequency vibrational modes to the peak frequency of 60 cm^{-1} in the water bulk to avoid unphysically large entropy contributions, videlicet, the lower frequency modes are reset to 60 cm^{-1}. [10, 11]

4. Grand-canonical (constant potential) calculations

Grand-canonical calculations were performed on the fully optimized structures in implicit solvent enabled by VASPsol. [12] And the grand free energy (Ω) at U is calculated as

$$\Omega(U) = G(U) + (3.61 + U_{\mathrm{fermi\,shift}} + U) \times (N_{\mathrm{e}} - N_{\mathrm{e0}})$$

where $U_{\mathrm{fermi\,shift}}$ is the correction for Fermi shift; $G(U)$ represents the Gibbs free energy under applied U; N_{e} and N_{e0} are the numbers of electrons at U and in the charge-neutral system, respectively (note that $N_{\mathrm{e}} = N_{\mathrm{e0}}$ for gas-phase and water molecules); the value of 3.61 is the absolute potential of RHE at pH = 14.

5. Dependence on the applied potential and the grand free energy profiles

From the previous studies,[10, 11] we found that the relative free energies (including barriers) are linearly dependent on U for $|U|$ <~2 V. This assumed linear relationship neglects the capacitive effect and was further justified as in Figure S15 and Table S6. Thus, we calculated the dependence on U assuming a linear relationship between U = 0.0 and + 2.0 V. Thus, we calculate the coefficient of U (a_U) as the following

$$a_U = \frac{(\Omega(U = 2\ \mathrm{V}) - \Omega(U = 0\ \mathrm{V}))}{2.00}$$

Thus, the $\Omega(U)$ between $U = 0$ to + 2 V can be calculated by

$$\Omega(U) = \Omega(0\ \mathrm{V}) + a_U \times U$$

Note that all the $\boldsymbol{\Omega}$ of states are referenced to $\boldsymbol{\Omega}$ of S0, so is the $\boldsymbol{a_U}$. And because of this, there is an additional electrochemical energy contribution from the number of electrons transferred starting from S0 as follows,

$$\Omega(\eta_0)(\mathrm{S}_i) = \Omega(\eta_0 = 0)(\mathrm{S}i) + a_U\eta_0 - n_e \times (\eta_0 + 1.23)$$

$$= \Omega(\eta_0 = 0)(\mathrm{S}i) - n_e \times 1.23 + a_U\eta_0 - n_e\eta_0 \equiv \Omega'(\eta_0 = 0)(\mathrm{S}i) + a_U\eta_0 - n_e\eta_0$$

Note that we group the first two terms marked in blue in the second line into a single term $\Omega'(\eta_0 = 0)(Si)$ for simplicity. $\Omega'(\eta_0 = 0)(Si)$ of all states are listed in Table S3, and the corresponding a_U are listed in Table S3 as well. n_e stands for the number of electrons transferred starting from S0, or the number of electrochemical steps between Si and S0.

The grand free energy change of any elementary step at specific η_0 can be simply calculated as

$$\Delta\Omega(\eta_0) = \Omega(\eta_0)(\mathrm{FS}) - \Omega(\eta_0)(\mathrm{IS})$$

and the barrier at specific U is,

$$\Delta\Omega^{\neq}(\eta_0) = \Omega(\eta_0)(\mathrm{TS}) - \Omega(\eta_0)(\mathrm{IS})$$

where IS, FS and TS represent the initial state, final state and transition state of the elementary step.

Supplementary Notes

1. On the use of GCE-TST

The classic transition state theory (TST) gives a simple form of $k_B T/h$ for the pre-exponential factor, and this has been shown to render quantitative predictions for heterogeneous catalysis by previous studies such as the classic work by Nørskov *et al.* [13] Recently, Melander *et al.* proved that this $k_B T/h$ pre-exponential factor also holds for the reactions at the electrochemical interfaces (open systems), which is the grand-canonical ensemble TST (GCE-TST) [14].

With the form of $k_{ij} = k_B T/h \cdot \exp(-\Delta\Omega^{\neq}/k_B T)$, the concentrations of non-adsorbed reactants (such as H_2O and OH^-), which determines their chemical potentials (free energies), are explicitly included in $\Delta\Omega^{\neq}$, so the pre-exponential factor is simply $k_B T/h$. Because the contributions from the concentrations to the free energy are entropic terms, we can write the above expression into a different form. Take $[OH^-]$ as an example (assuming OH^- is a reactant), its contribution to the free energy barrier can be singled out as $\Delta\Omega^{\neq} = \Delta\Omega^{\neq *} - k_B T \ln[\mathrm{OH}^-]$, where $\Delta\Omega^{\neq *}$ is simply the free energy barrier without including the contribution from $[OH^-]$. Consequently, the expression of the rate constant can be written as $k_{ij} = k_B T/h \cdot [\mathrm{OH}^-] \cdot \exp(-\Delta\Omega^{\neq *}/k_B T)$, and the pre-exponential factor now becomes $k_B T/h \cdot [\mathrm{OH}^-]$, which explicitly includes the concentration of OH^-. The two different forms are equivalent, and we used the first one with the simple $k_B T/h$ pre-exponential factor in this work. Nevertheless, we note that the pre-exponential factor in electrochemistry can be complicated by various factors at the electrochemical interfaces[15], which thus needs further theoretical developments.

Table S1. Optimized lattice constants of Ni(M)OOH (M = Fe, Co, Ni) slab supercells.

Composition	Lattice constants / Å		
	a	*b*	*c* (Fixed)
NiOOH	11.20	4.87	25.00
Ni(Co)OOH	11.29	4.92	25.00
Ni(Fe)OOH	11.31	4.88	25.00

Table S2. Calculated free energies of species in the OER equation.

Species	Pressure / bar	Temperature / K	G / eV (pH = 0)	Ω / eV (U = 0, pH = 14)
O_2 (*g*)	1.000	298.15	−9.921	−9.921
H_2 (*g*)	1.000	298.15	−6.800	−8.458
H_2O (*l*)	0.031	298.15	−14.220	−14.220

Table S3. Grand free energy at $\eta_0 = 0$ $(\Omega'(\eta_0 = 0))$ in eV and coefficient of U (a_U) in e for each state. Note that all grand free energies are referenced to that of S0, and S00 is the same state as S0 but with a whole cycle of OER finished.

State	n_e	NiOOH		Ni(Co)OOH		Ni(Fe)OOH	
		$\Omega'(\eta_0 = 0)$	a_U	$\Omega'(\eta_0 = 0)$	a_U	$\Omega'(\eta_0 = 0)$	a_U
S0	0	0	0	0	0	0	0
S1	1	0.2855	+ 0.0031	0.4021	+ 0.2868	0.1572	+ 0.1229
S2	2	1.0237	+ 0.1030	0.9677	+ 0.2674	0.5250	+ 0.0157
S3	2	0.5627	− 0.2638	1.1038	− 0.0547	0.5257	− 0.0162
S4	3	0.8783	− 0.0952	0.8622	− 0.0928	0.0761	− 0.0725
S5	1	0.8498	− 0.3967	0.2836	− 0.1735	1.3537	− 0.0167
S6	2	1.0622	+ 0.3233	0.6764	− 0.0263	0.7079	+ 0.0585
S7	1	− 0.0642	− 0.3412	0.9319	− 0.3218	0.7192	− 0.0569
S8	2	− 0.5955	− 0.4179	0.6408	+ 0.0579	0.8516	− 0.0776
S9	3	− 0.4812	− 0.2920	0.2186	− 0.2684	0.1669	− 0.0968
S00	4	0	0	0	0	0	0
Transition states							
AEM	2	1.7635	− 0.2033	1.5231	− 0.3964	1.1475	− 0.0807
IMOCa	1	1.4305	+ 0.1370	1.9791	+ 0.0766	1.7928	+ 0.1119
IMOCb	2	2.2441	+ 0.2032	1.5153	+ 0.2267	1.5446	+ 0.1164
LOMa	1	1.2712	+ 0.1465	1.4173	+ 0.1658	0.9309	+ 0.0781
LOMb	2	1.7715	− 0.0100	1.6655	+ 0.1348	1.3906	+ 0.2373

Table S4. Changes in the predicted overpotential ($\Delta\eta$) induced by the introduced deviation (ε) of ± 0.1 eV in the free energy of each state to mimic the uncertainty in DFT-calculated energetics. (Note that "N. A." marks the states that are not present in the PLS scheme.)

State	Microkinetic modeling scheme		PLS scheme	
	$\varepsilon = +0.10$ eV	$\varepsilon = -0.10$ eV	$\varepsilon = +0.10$ eV	$\varepsilon = -0.10$ eV
		$\Delta\eta$ (V)		
S0	0	0	0	0
S1	−0.08	0.08	−0.10	0.10
S2	0	0.01	0.10	−0.10
S3	0	0	N. A.	N. A.
S4	0	0	0	0
S5	0	0	0	0
S6	0	0	0	−0.07
S7	0	0	N. A.	N. A.
S8	0	0	0	0
S9	0	0	0	0
TS_AEM	0.07	−0.08	N. A.	N. A.
TS_IMOCa	0	0	N. A.	N. A.
TS_IMOCb	0	0	N. A.	N. A.
TS_LOMa	0	0	N. A.	N. A.
TS_LOMb	0	−0.03	N. A.	N. A.

Table S5. Total magnetic moments (μ_B) for all states at different potentials.

	NiOOH		Ni(Co)OOH		Ni(Fe)OOH	
	U = 0	U = 2 V	U = 0	U = 2 V	U = 0	U = 2 V
S0	7.33	7.28	10.82	10.69	17.16	16.78
S1	7.58	7.80	8.53	8.33	19.74	19.75
S2	7.69	8.74	9.96	10.57	18.95	18.79
S3	7.37	8.66	8.69	8.82	18.70	18.85
S4	7.60	8.18	9.84	9.13	19.70	19.95
S5	8.97	8.10	7.74	8.11	21.52	22.09
S6	8.21	8.31	8.89	11.00	14.17	14.84
S7	8.07	8.10	8.70	10.71	19.66	19.89
S8	9.18	8.83	8.91	12.99	22.47	22.92
S9	8.12	8.02	9.63	7.94	19.58	19.92

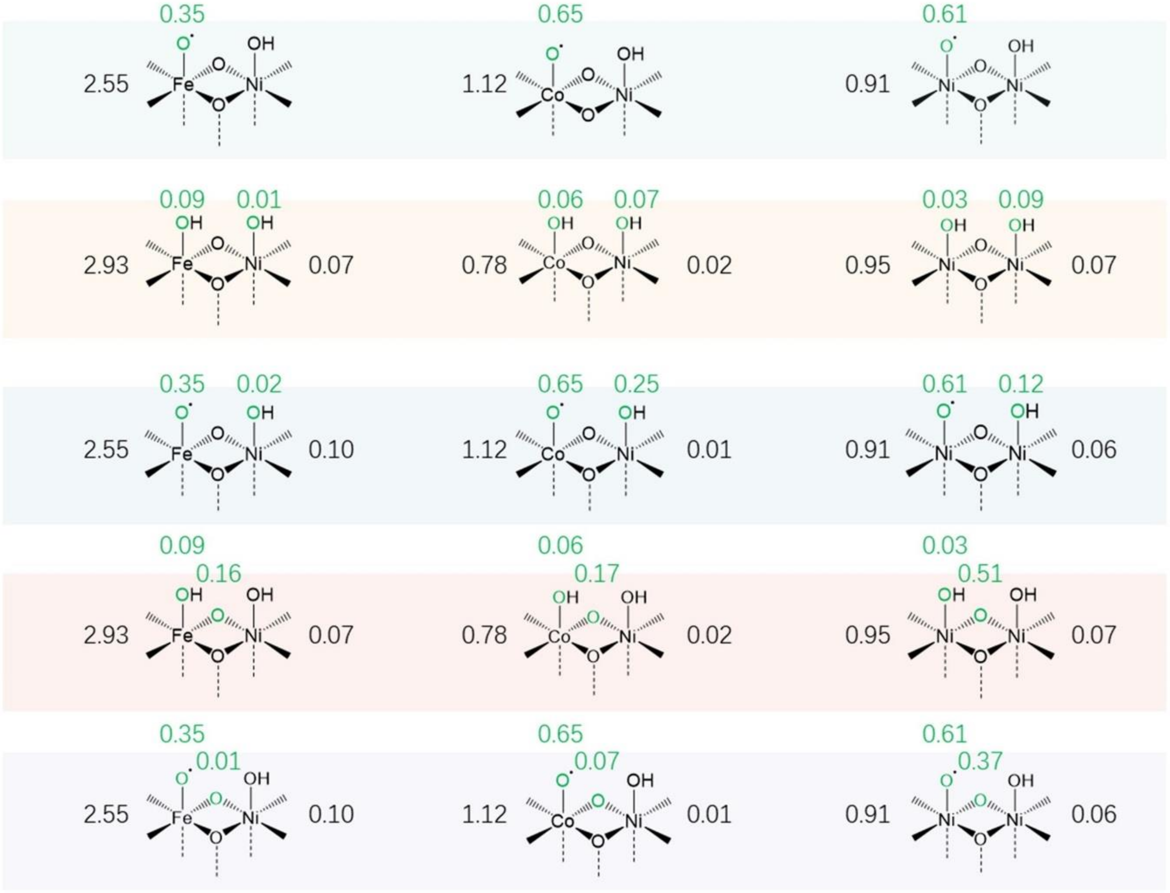

Figure S1. Spin densities on the metal sites and reacting O species in the states before O-O coupling in the AEM, IMOCa, IMOCb, LOMa and LOMb pathways on Ni(M)OOH, M = Ni, Co, and Fe.

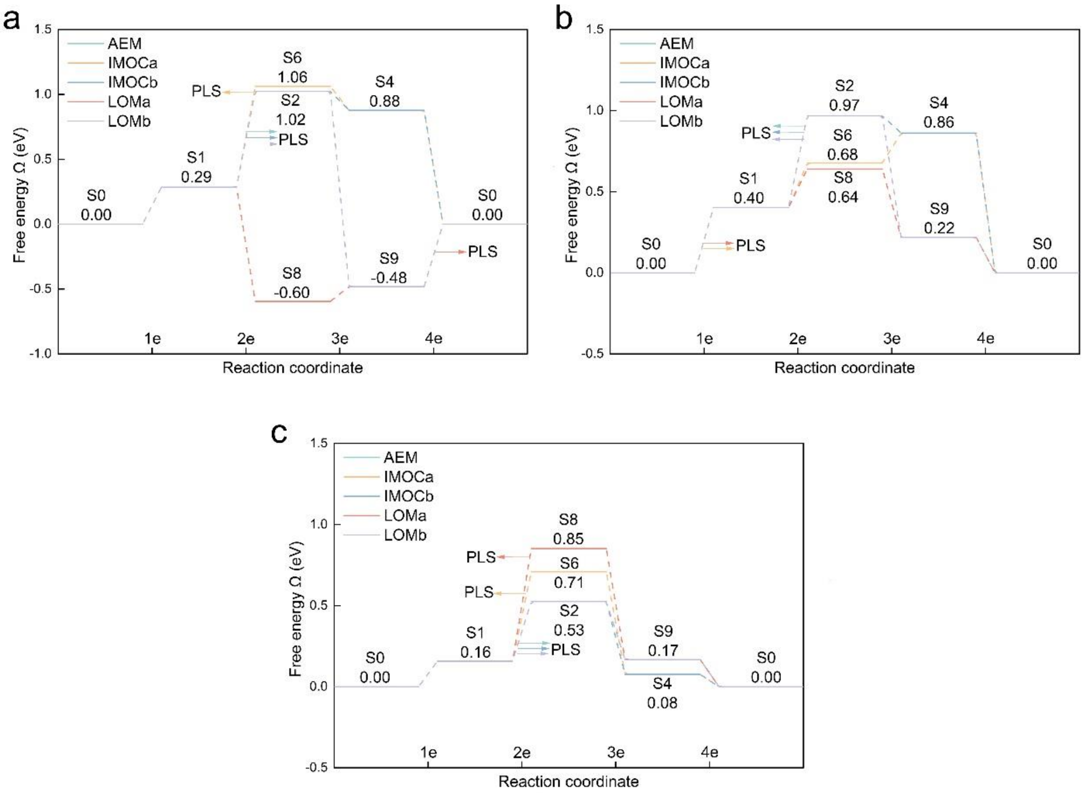

Figure S2. PLS scheme at U = 1.23 V for OER on (a) NiOOH, (b) Ni(Co)OOH, and (c) Ni(Fe)OOH following different reaction mechanisms.

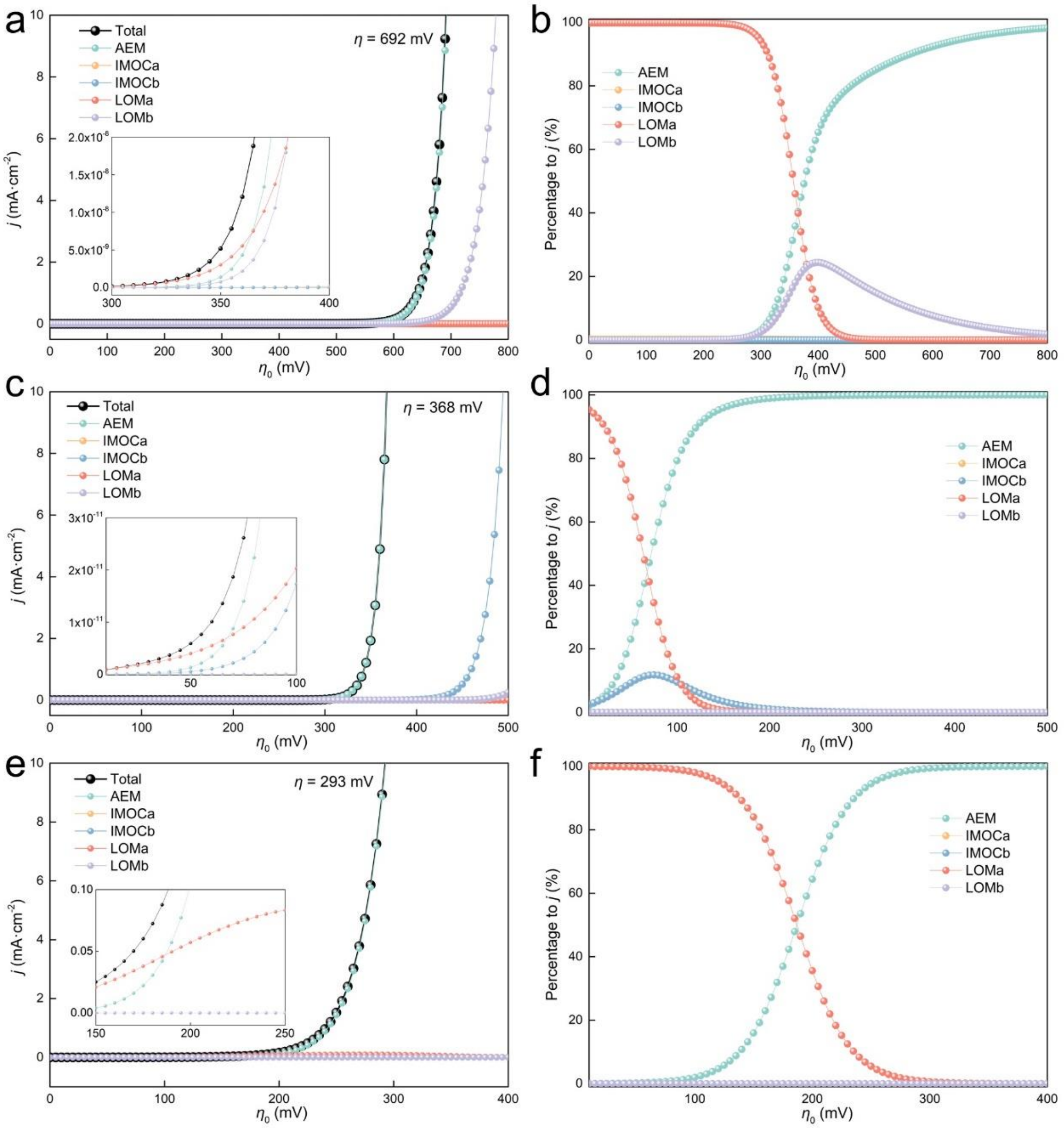

Figure S3. Calculated current densities (j) as functions of the applied potential and percentage of individual contribution from each mechanism to the total j from the microkinetic modeling on the coupled reaction network on (a,b) NiOOH, (c,d) Ni(Co)OOH, and (e,f) Ni(Fe)OOH.

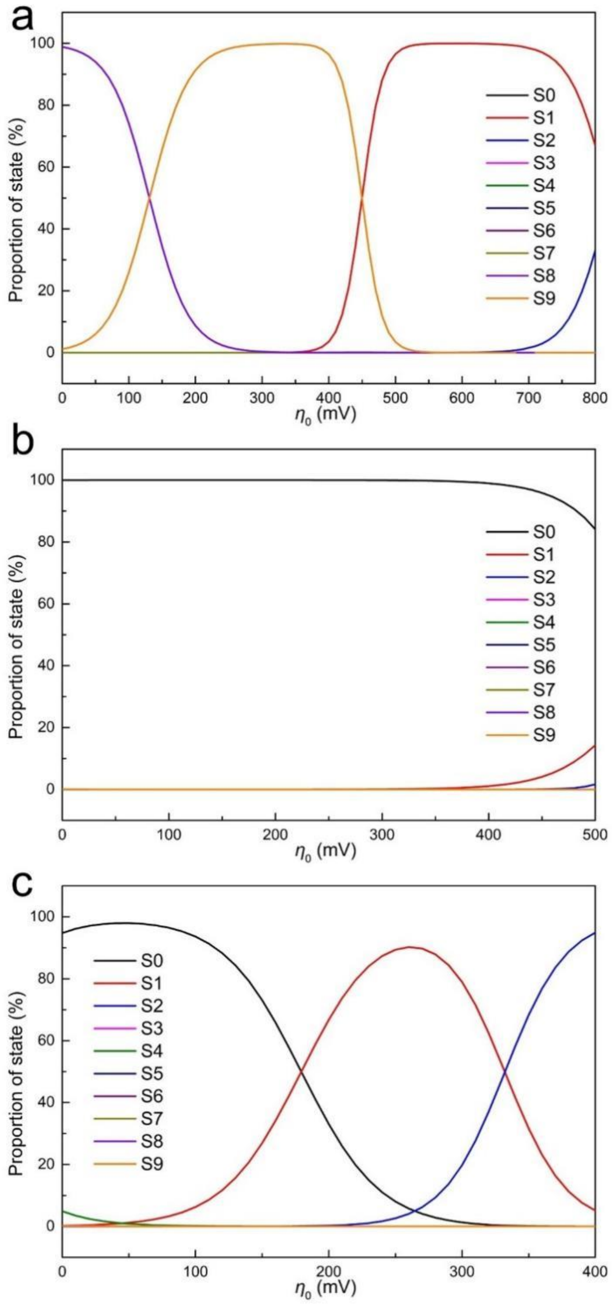

Figure S4. (a-c) Proportion of state S*i* as a function of applied potential η_0 on Ni(M)OOH (M = Ni, Co, Fe). All these proportions will emerge naturally in our microkinetic modeling, and are independent of the choices of so-called initial states, because they form catalytic cycles that have no starting points to define. Nevertheless, we take S0 as the so-called initial state for all cases, because it has a bare M site, which can be regarded as a starting point by convention.

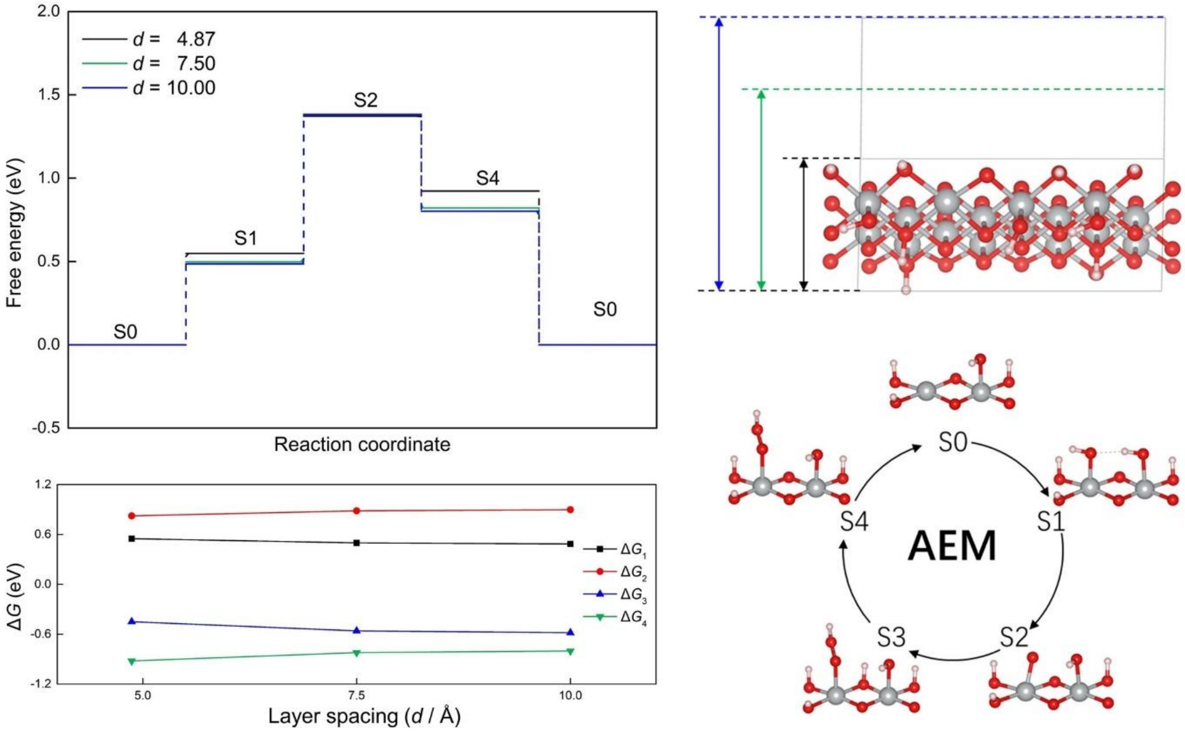

Figure S5. Convergence of reaction free energies of electrochemical steps in AEM of OER on NiOOH with respect to varying layer spacing (d / Å) from 4.87 Å (the equilibrium spacing) to 10 Å. The changes in the reaction free energies are all within 0.13 eV, even when the layer spacing is doubled.

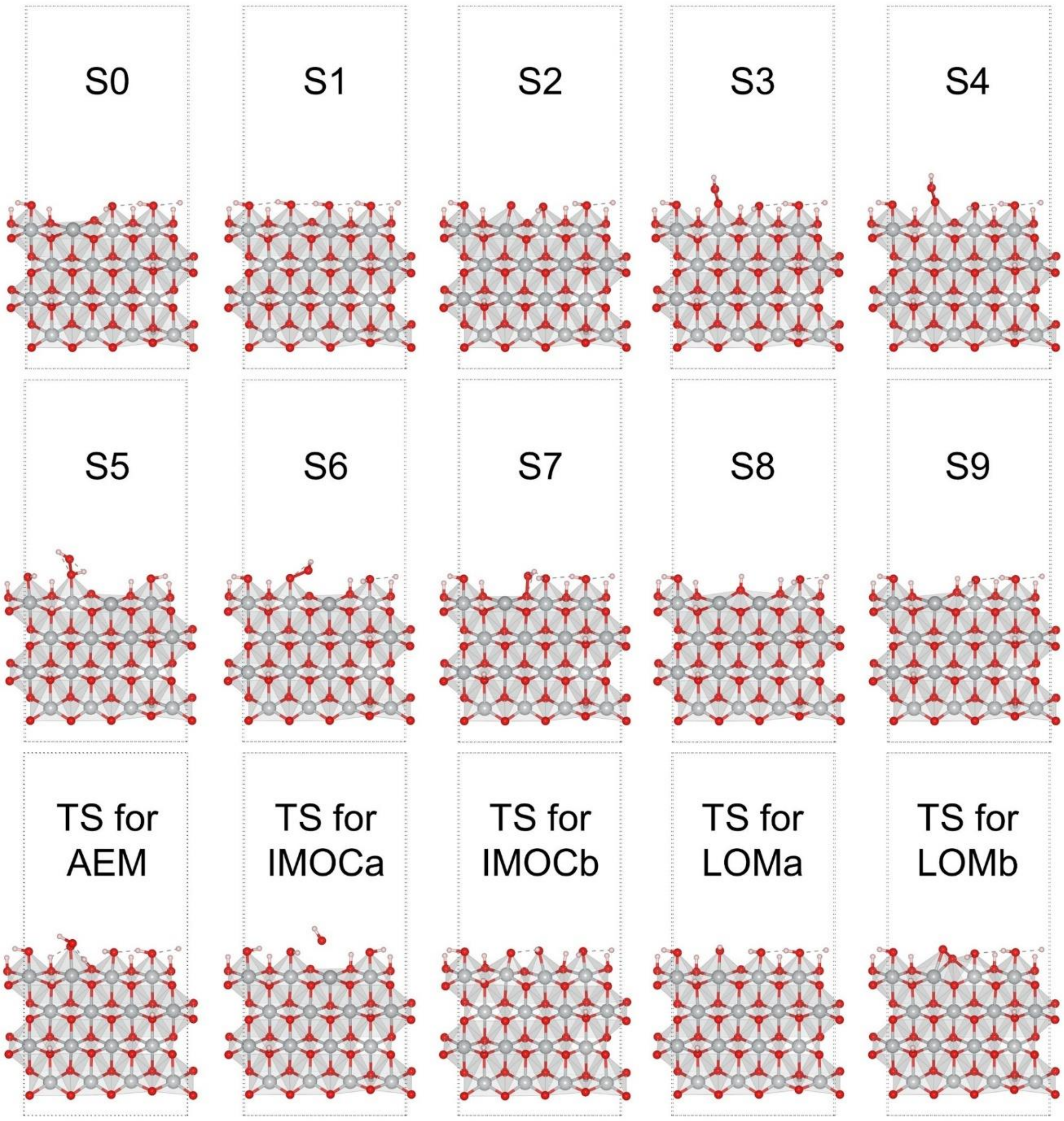

Figure S6. The optimized structures of all states for OER on NiOOH.

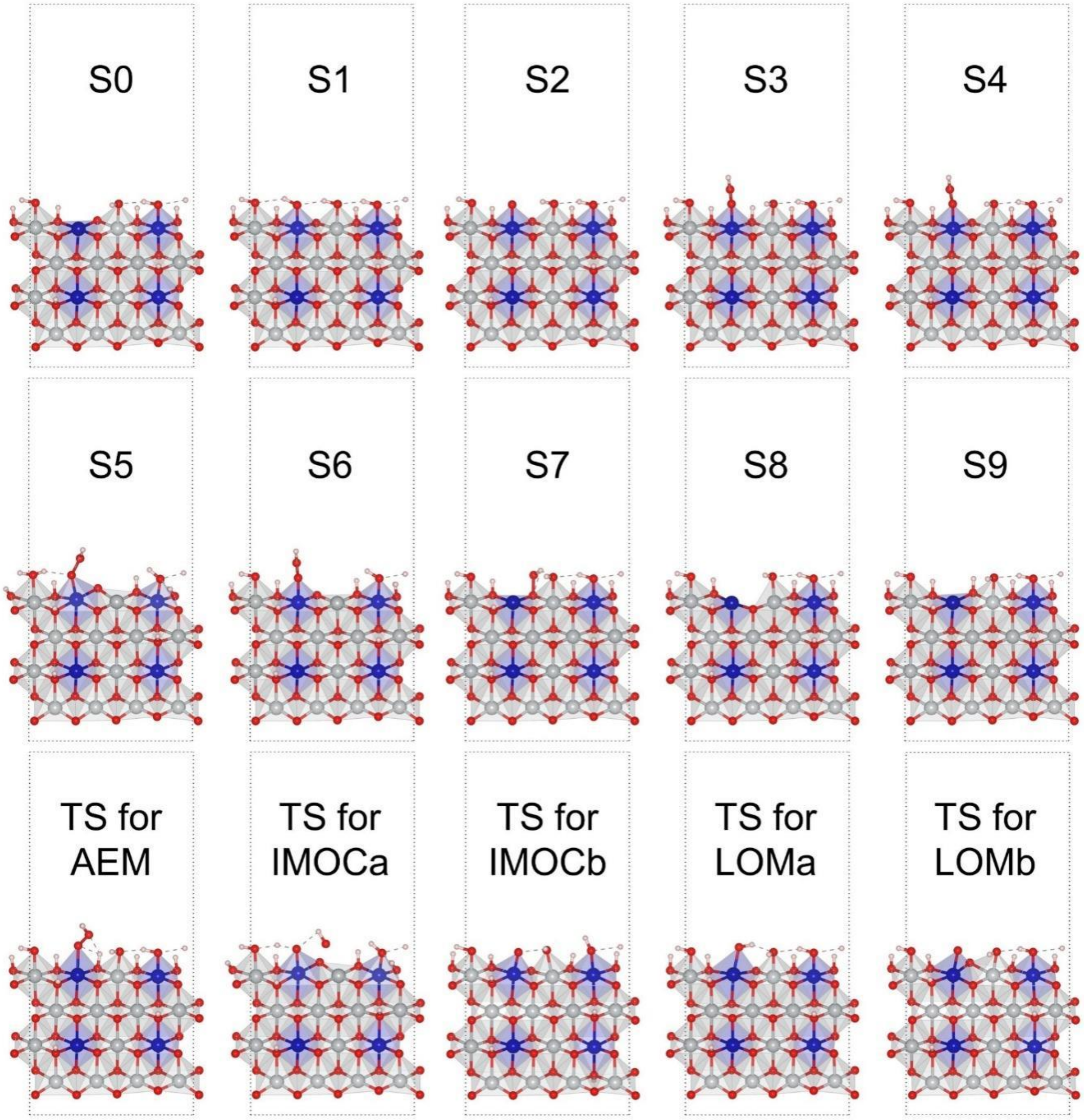

Figure S7. The optimized structures of all states for OER on Ni(Co)OOH.

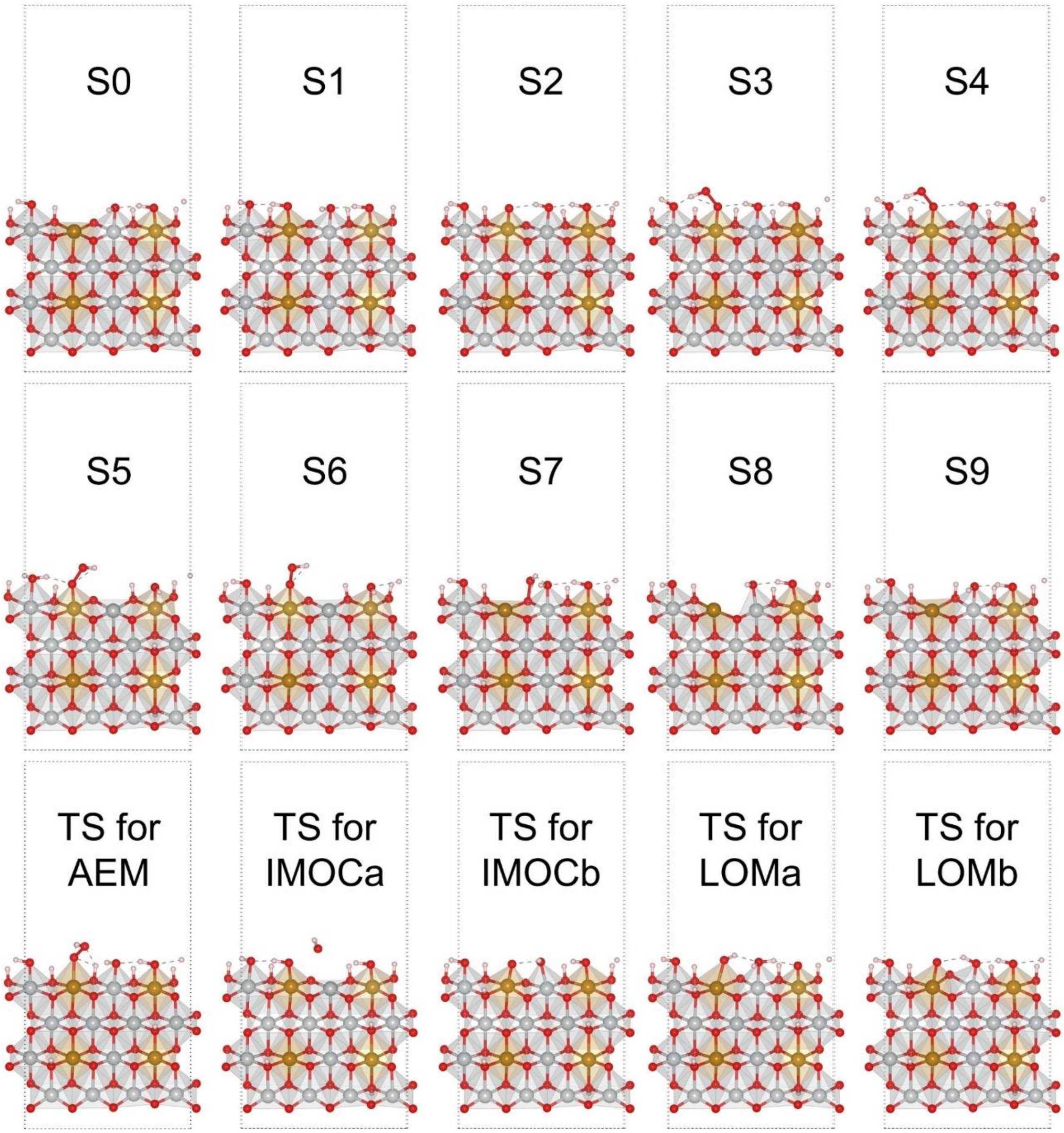

Figure S8. The optimized structures of all states for OER on Ni(Fe)OOH.

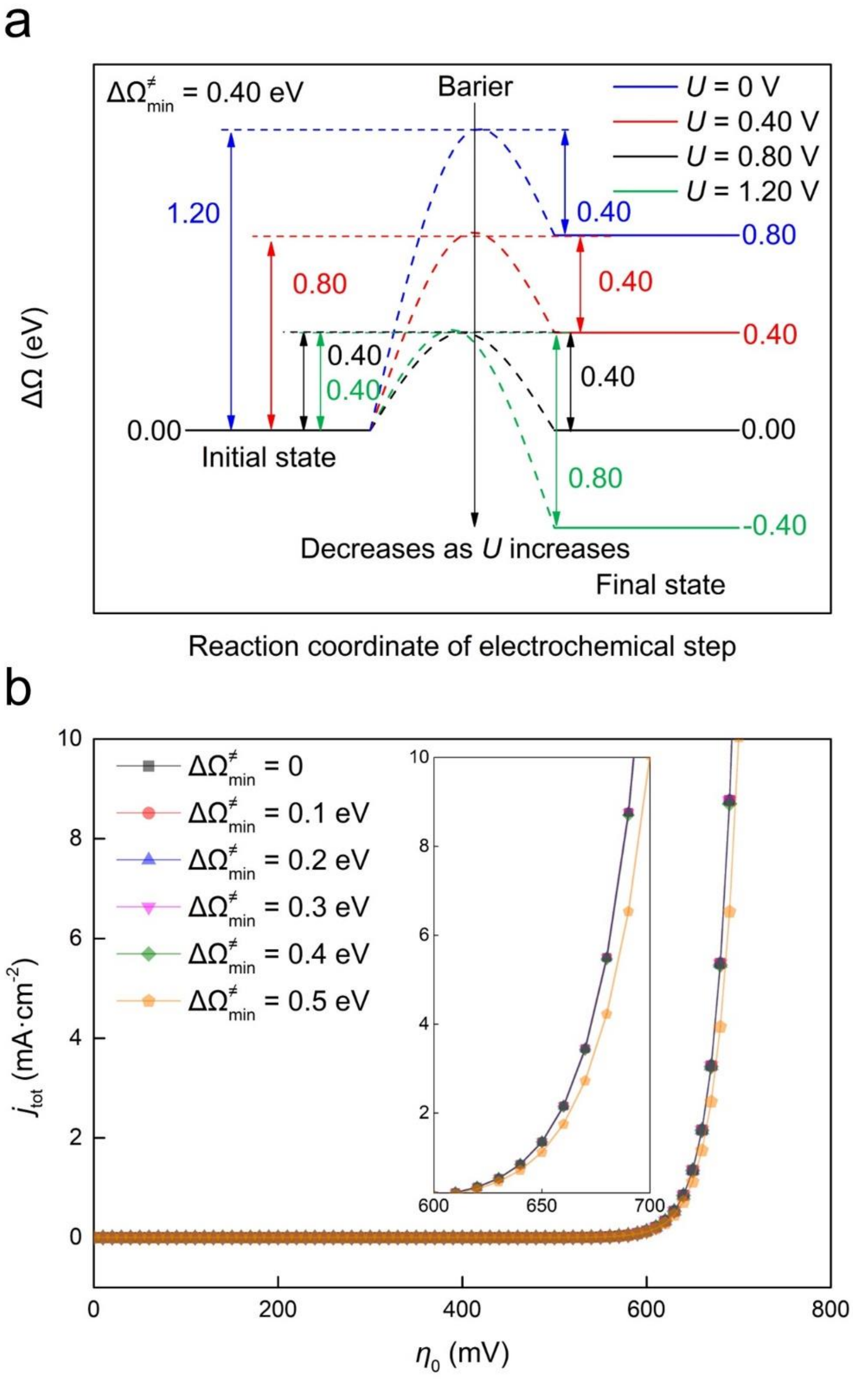

Figure S9. (a) Illustration of the rigid minimal barrier ($\Delta\Omega^{\neq}_{\rm min}$) of 0.40 eV we impose on the barrier of electrochemical step with $\Delta\Omega$ = 0 eV at U = 0.8 V. The barrier ($\Delta\Omega^{\neq}$) of electrochemical step is related to $\Delta\Omega^{\neq}_{\rm min}$ and the reaction free energy ($\Delta\Omega$) *via* the expression below,

$$\Delta\Omega^{\neq} = \begin{cases} \Delta\Omega^{\neq}_{\min}, & \text{for } \Delta\Omega \leq 0 \\ \Delta\Omega^{\neq}_{\min} + \beta \cdot \Delta\Omega & \text{for } \Delta\Omega > 0 \end{cases}$$

So, $\Delta\Omega^{\neq}_{\min}$ sets the lower bound for the barrier when $\Delta\Omega \leq 0$ and becomes an additional rigid term to the barrier when $\Delta\Omega > 0$. In order to test the most extreme scenario, we chose $\boldsymbol{\beta = 1}$ here, because this gives the fastest increase in $\Delta\Omega^{\neq}$ as $\Delta\Omega$ increases (when $\Delta\Omega > 0$). (Note that β is typically ~0.5, so we also performed a test with $\beta = 0.5$ in Figure S16 that gives the same conclusions.) It is worth noting that, with including $\Delta\Omega^{\neq}_{\min}$, the barriers of electrochemical steps can be larger than those for the O-O coupling steps at low potentials. Thus, the O-O coupling steps are not assumed to be the rate limiting step, even with $\Delta\Omega^{\neq}_{\min}$ = 0 eV. At high potentials, the barriers of electrochemical steps decrease significantly, and effectively, the O-O coupling may become the rate limiting step. (b) Current density as the function of overpotential η_0 with different rigid minimal barrier ($\Delta\Omega^{\neq}_{\min}$) imposed on the electrochemical steps in OER on NiOOH. Note that the current density and thus the catalytic performance has little dependence on the $\Delta\Omega^{\neq}_{\min}$ of electrochemical step up to 0.4 eV. If barriers of electrochemical steps could survive to be 0.50 eV at high applied potentials (supposedly to be reduced to small values), then the catalytic performance would be influenced by them to a noticeable extent.

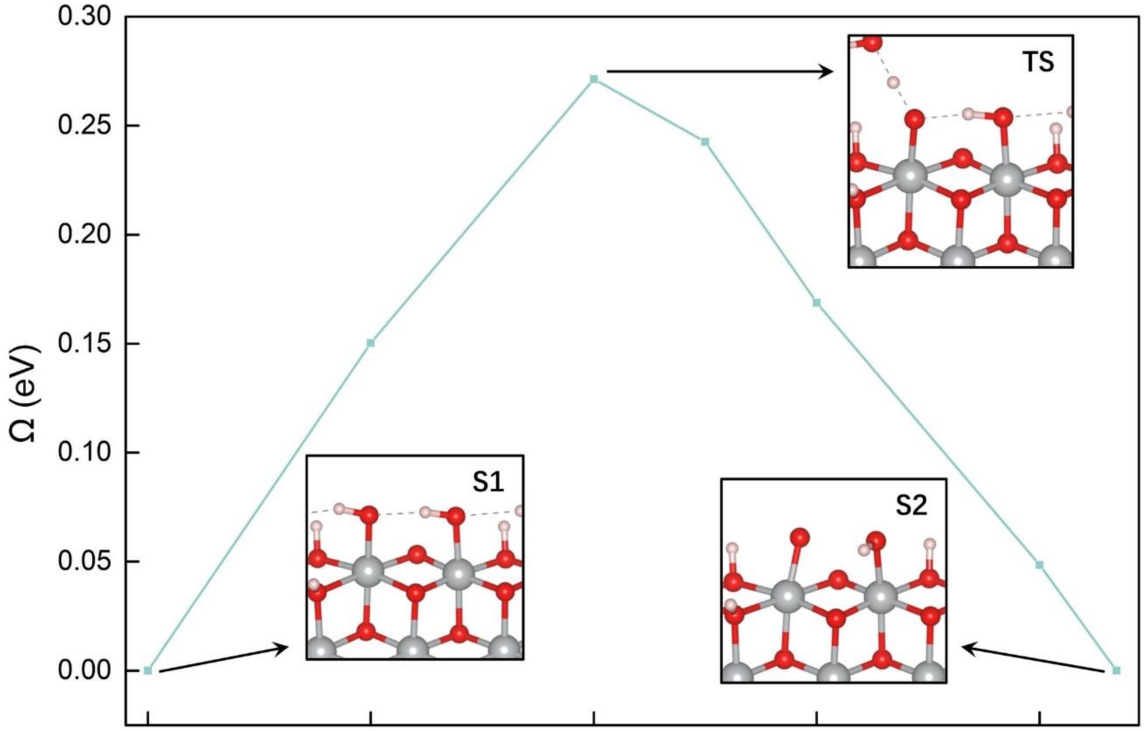

Figure S10. The barrier for the electrochemical step from **S1** to **S2** on NiOOH (*OH $\rightarrow$ *O·) at $\mathbf{\Delta\Omega} = 0$ eV is calculated to be 0.27 eV, which is equivalent to possessing a $\Delta\Omega^{\neq}_{\mathrm{min}}$ = 0.27 eV.

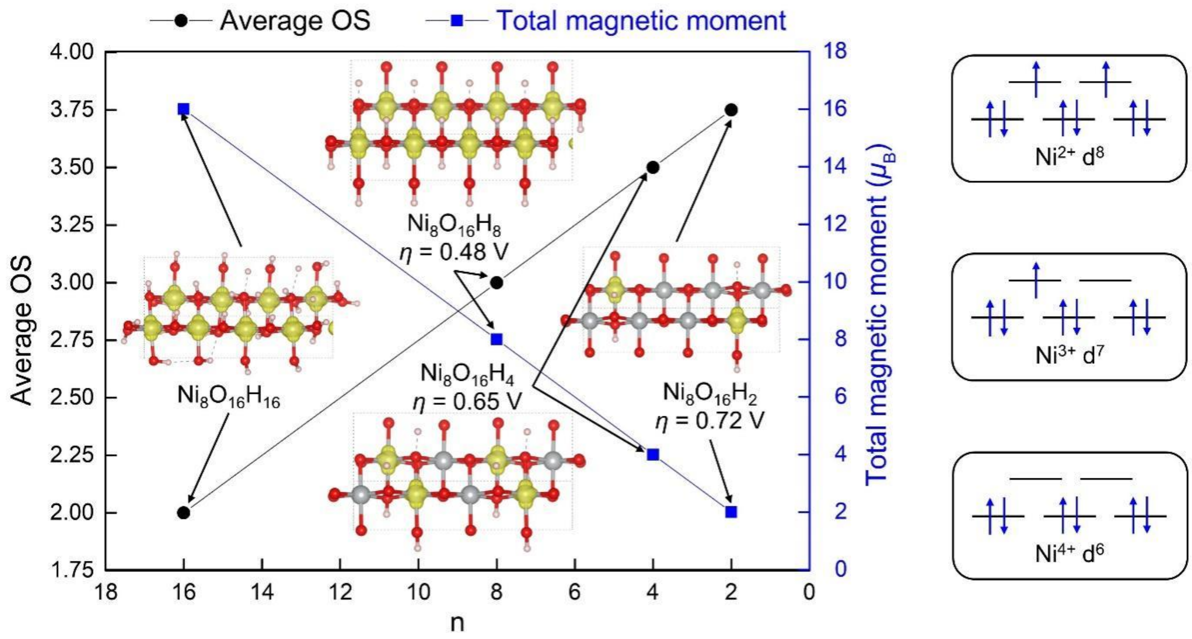

Figure S11. The average oxidation state (OS) and the total magnetic moment of gradually deprotonated NiOOH with n denoting the number of H atoms and η the corresponding overpotential. The insets are the spin density contours of the systems. The electron configurations of Ni^{2+}, Ni^{3+}, and Ni^{4+} are shown in the right panel, which indicate their numbers of unpaired electron(s).

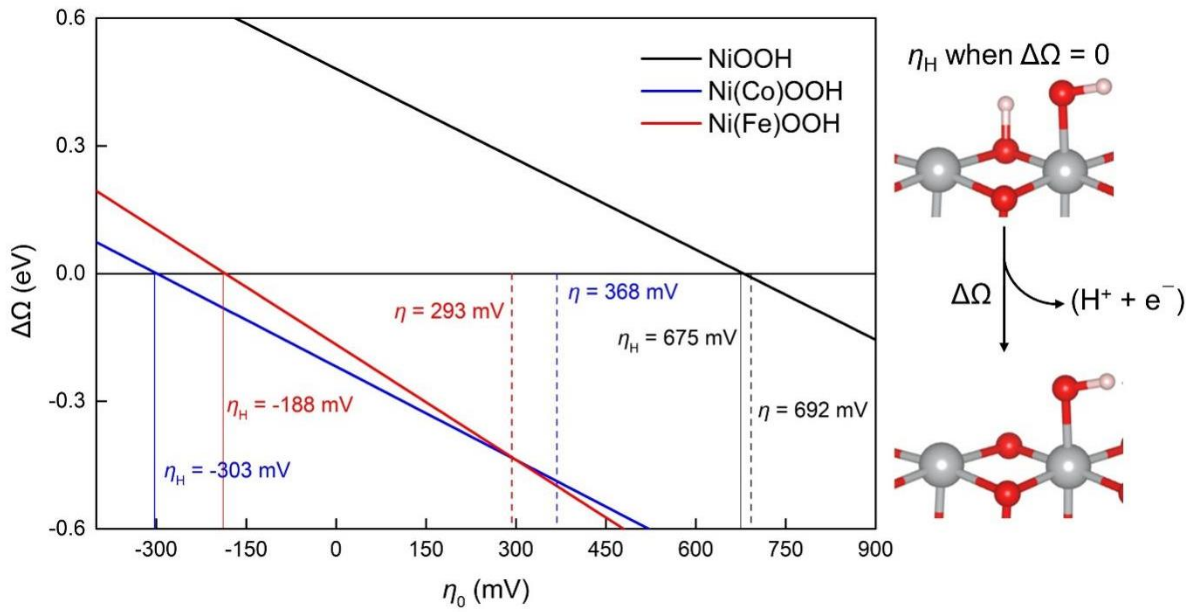

Figure S12. The grand-canonical free energy changes (ΔΩ) for the electrochemical deprotonation (oxidation) of the surface models to reach the average OS of +3.75. Note that η_{H} marks the applied potential when $\Delta\Omega = 0$, and all η_{H} are lower than the corresponding η (the working potential for OER on each catalyst), so it is exothermic for all surfaces to reach the average OS of +3.75 at η.

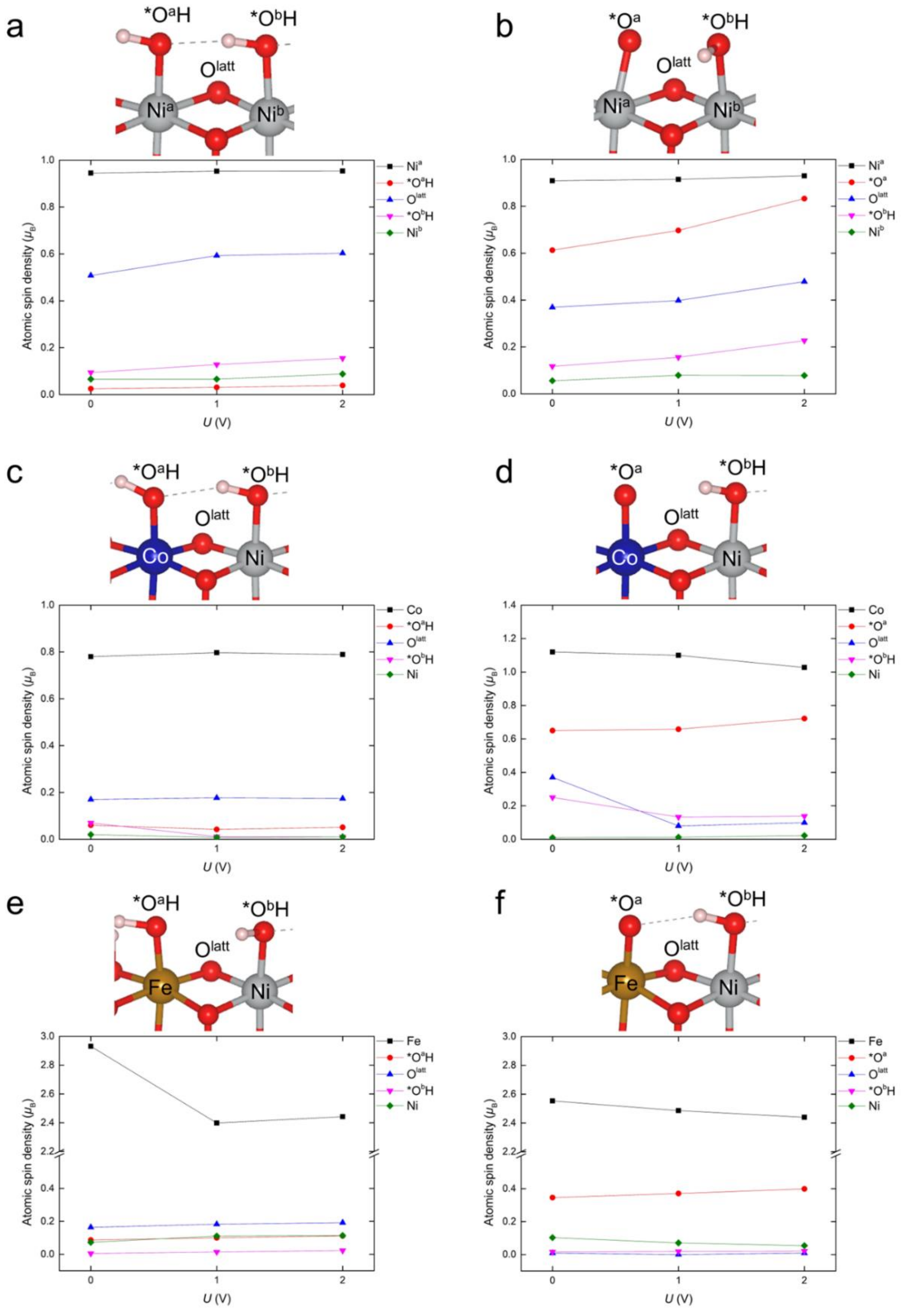

Figure S13. Atomic spin densities of catalytically active metal sites and reacting O species in the states of S1 and S2 for (a, b) NiOOH, (c, d) Ni(Co)OOH, and (e, f) Ni(Fe)OOH under different potentials.

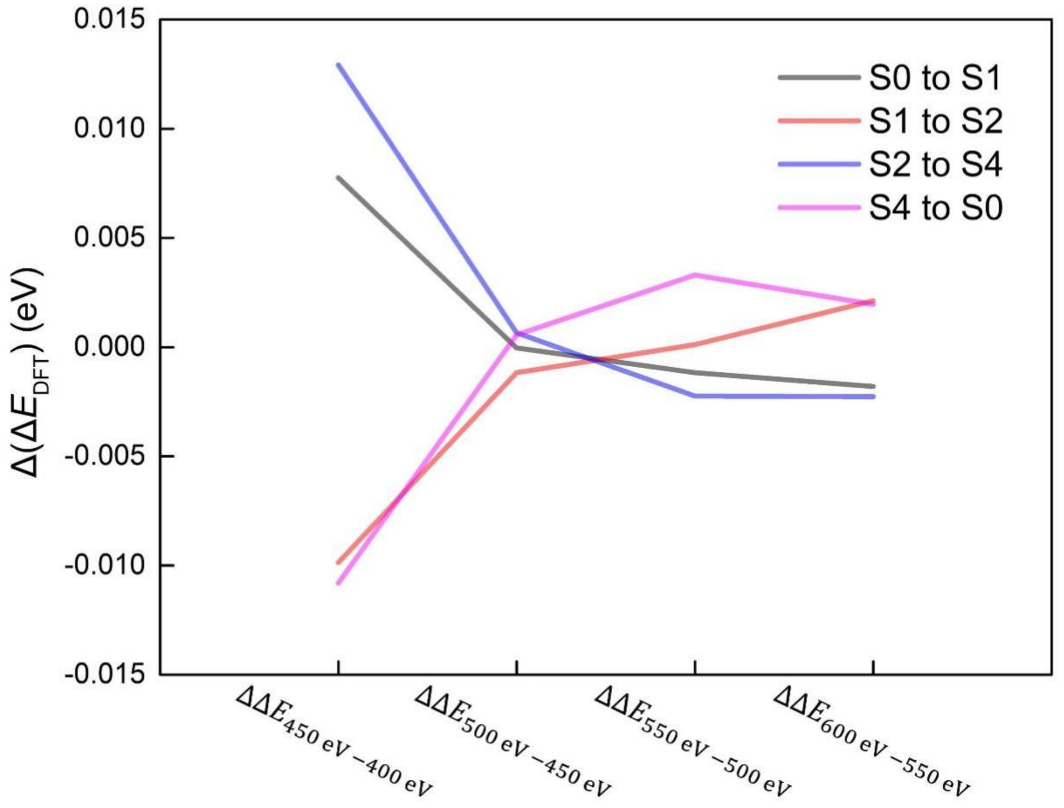

Figure S14. Benchmark calculations on the cut-off energy with all the reaction energies in AEM on NiOOH. This shows that the cut-off energy of 500 eV is sufficient to converge the reaction energies to within 0.005 eV, which is less than the typical uncertainty in the DFT-calculated energetics.

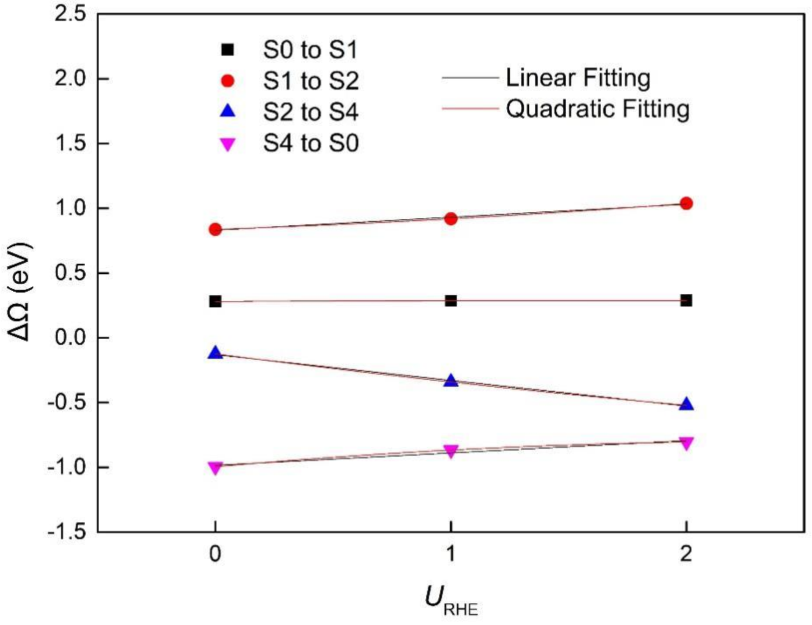

Figure S15. Linear and quadratic fitting of $\Delta\Omega(U)$ for all the reaction energies in AEM on NiOOH. The two types of fitting render essentially overlapping curves, and Table R2 lists the fitted parameters.

Table S6. Fitted parameters for the linear fitting with the formula $\Delta\Omega = b_0 + b_1 U$ and the quadratic fitting with the formula $\Delta\Omega = b_0 + b_1 U + b_2 U^2$. The small b_2 values imply that the capacitive effect is negligible here. The last column lists the differences between $\Delta\Omega$ values (in eV) at U = 1 V calculated from the two types of fitting, all of which are small.

Steps	Linear Fitting		Quadratic Fitting			Difference
	b_0	b_1	b_0	b_1	b_2	at U = 1 V
S0 to S1	0.282	0.003	0.282	0.009	-0.003	0.002
S1 to S2	0.831	0.100	0.837	0.064	0.018	-0.012
S2 to S4	-0.130	-0.198	-0.124	-0.208	0.005	0.001
S4 to S0	-0.984	0.095	-0.995	0.136	-0.020	0.009

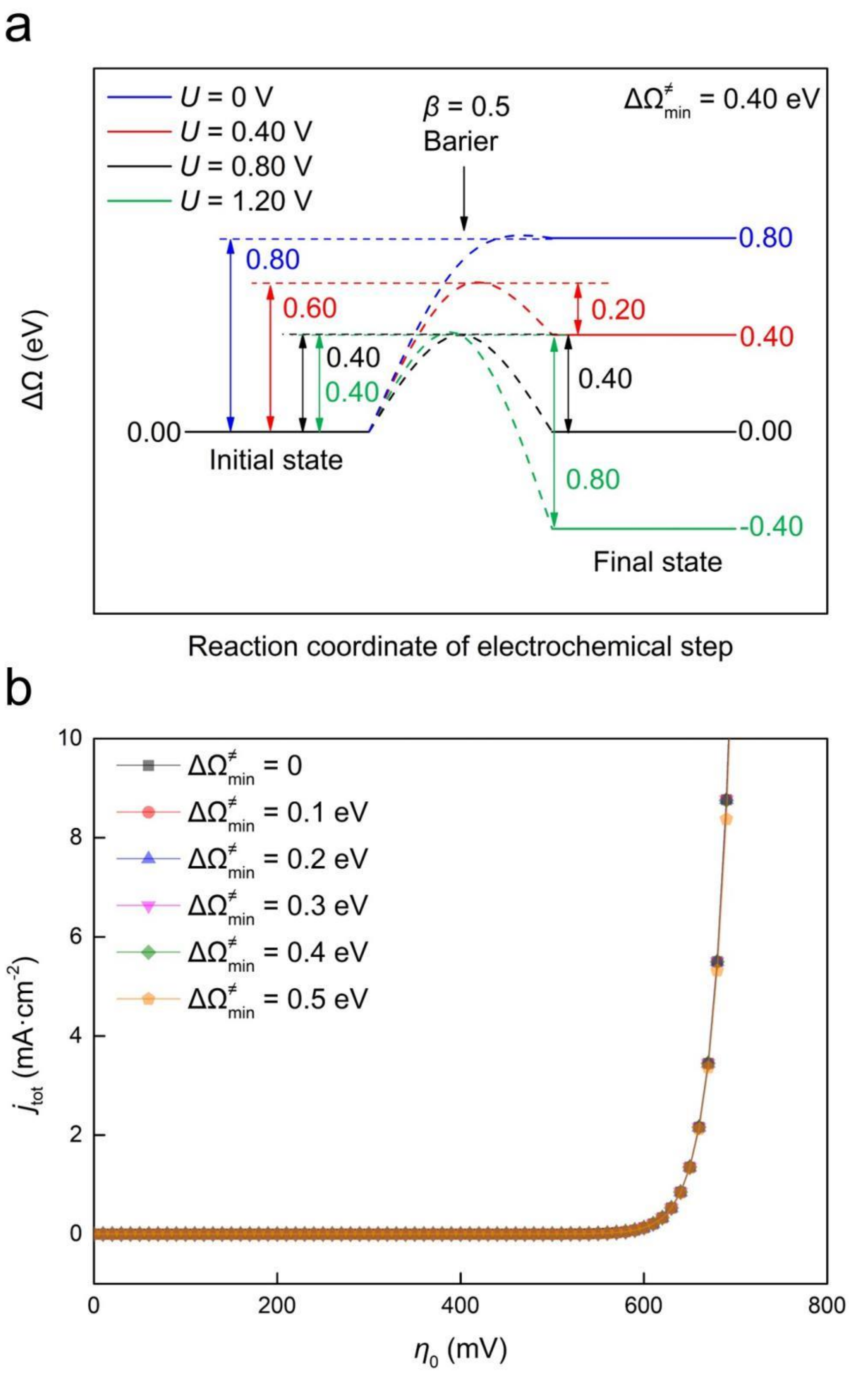

Figure S16. (a) Illustration of the rigid minimal barrier ($\Delta\Omega^{\neq}_{\min}$) of 0.40 eV we impose on the barrier of electrochemical step with $\Delta\Omega = 0$ eV at $U = 0.8$ V. The barrier ($\Delta\Omega^{\neq}$) of electrochemical step is related to $\Delta\Omega^{\neq}_{\min}$ and the reaction free energy ($\Delta\Omega$) *via* the expression below,

$$\Delta\Omega^{\neq} = \begin{cases} \Delta\Omega^{\neq}_{\min}, & \text{for } \Delta\Omega \leq 0 \\ \Delta\Omega^{\neq}_{\min} + \beta \cdot \Delta\Omega & \text{for } \Delta\Omega > 0 \end{cases}$$

So, $\Delta\Omega^{\neq}_{\mathrm{min}}$ sets the lower bound for the barrier when $\Delta\Omega \leq 0$ and becomes an additional rigid term to the barrier when $\Delta\Omega > 0$. Here we test the typical $\boldsymbol{\beta}$ = **0.5**. (b) Current density as the function of overpotential η_0 with different rigid minimal barrier ($\Delta\Omega^{\neq}_{\mathrm{min}}$) imposed on the electrochemical steps in OER on NiOOH. Note that the current density and thus the catalytic performance has little dependence on the $\Delta\Omega^{\neq}_{\mathrm{min}}$ of electrochemical step up to 0.5 eV.

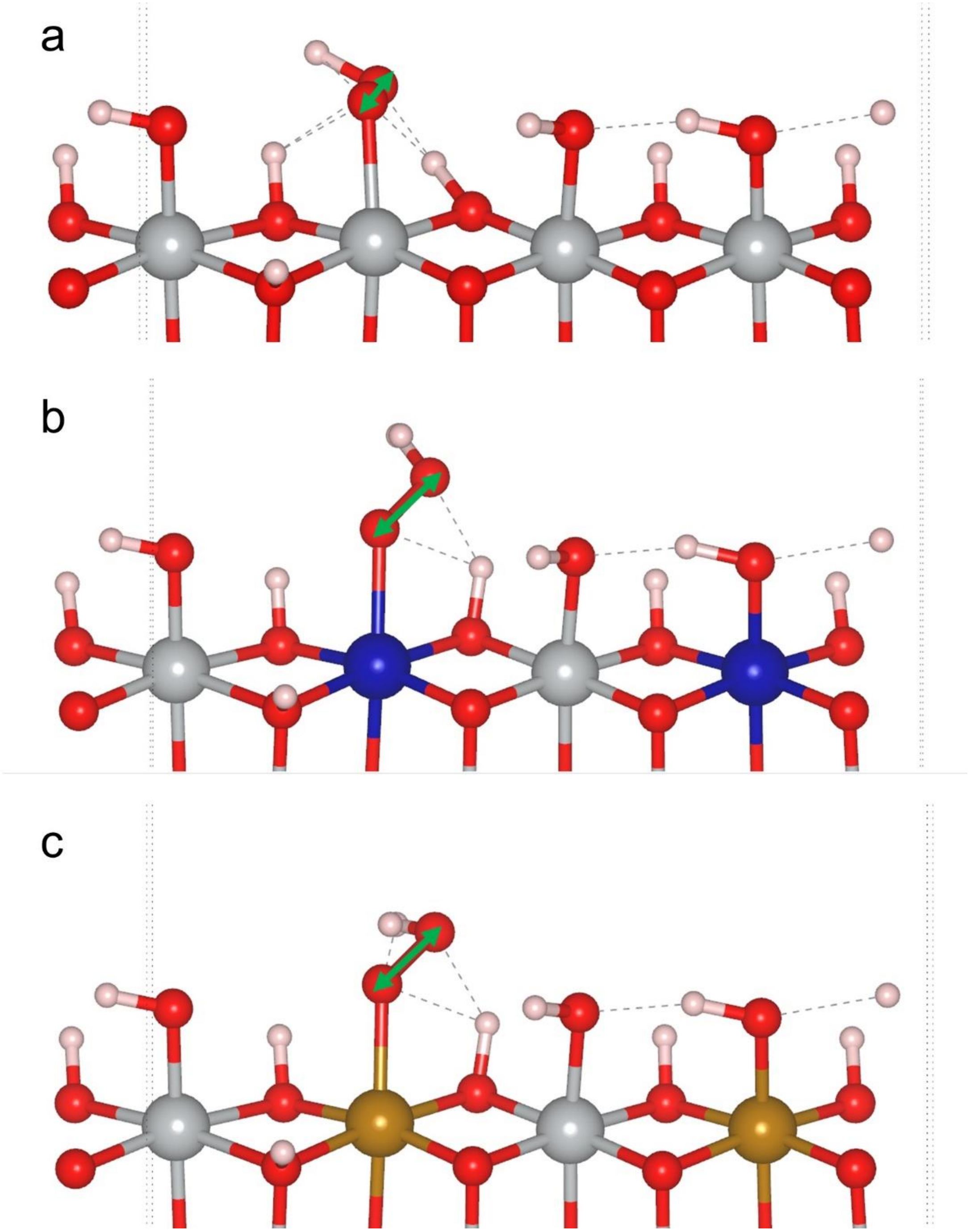

Figure S17. The transition states (TSs) of O-O coupling in AEM on (a) NiOOH, (b) Ni(Fe)OOH, and (c) Ni(Co)OOH. The green arrow marks the imaginary vibrational mode in each TS. This indicates that the O-O coupling started with OH^- (the OH^- pathway) might share the same TS as that started with H_2O (the H_2O pathway), although the OH^- pathway should have an additional

intermediate state with the bridging O site protonated. However, we argue that it is less likely for OH^- to serve as the reactant even at pH = 14, because the concentration of OH^- (1 M) is overwhelmed by that of H_2O (56 M), which is likely to result in a worse kinetics for the OH^- pathway assuming the same TS for the two pathways. If we included both pathways, the microkinetic modeling should be able to discern the two and predict the dominant one, which is likely to be the H_2O pathway.

Supplementary References

1. Van der Ven, A., Morgan, D., Meng, Y. S.&Ceder, G. Phase stability of nickel hydroxides and oxyhydroxides. *J. Electrochem. Soc.* **153**, A210-A215 (2006).
2. Goldsmith, Z. K., *et al.* Characterization of NiFe oxyhydroxide electrocatalysts by integrated electronic structure calculations and spectroelectrochemistry. *Proc. Natl. Acad. Sci. U.S.A.* **114**, 3050-3055 (2017).
3. Bediako, D. K., *et al.* Structure-Activity Correlations in a Nickel-Borate Oxygen Evolution Catalyst. *J. Am. Chem. Soc.* **134**, 6801-6809 (2012).
4. Li, L. F., Li, Y. F. & Liu, Z. P. Oxygen Evolution Activity on NiOOH Catalysts: Four-Coordinated Ni Cation as the Active Site and the Hydroperoxide Mechanism. *ACS Catal.* **10**, 2581-2590 (2020).
5. Zhang, B., *et al.* Homogeneously dispersed multimetal oxygen-evolving catalysts. *Science* **352**, 333-337 (2016).
6. Bajdich, M., Garcia-Mota, M., Vojvodic, A., Norskov, J. K. & Bell, A. T. Theoretical investigation of the activity of cobalt oxides for the electrochemical oxidation of water. *J. Am. Chem. Soc.* **135**, 13521-13530 (2013).
7. Dionigi, F.&Strasser, P. NiFe-Based (Oxy)hydroxide Catalysts for Oxygen Evolution Reaction in Non-Acidic Electrolytes. *Adv. Energy Mater.* **6**, 1600621 (2016).
8. Dionigi, F., *et al.* In-situ structure and catalytic mechanism of NiFe and CoFe layered double hydroxides during oxygen evolution. *Nat. Commun.* **11**, 2522 (2020).
9. Garcia-Mota, M., *et al.* Importance of Correlation in Determining Electrocatalytic Oxygen Evolution Activity on Cobalt Oxides. *J. Phys. Chem. C* **116**, 21077-21082 (2012).
10. Xiao, H., Cheng, T., Goddard, W. A., 3rd&Sundararaman, R. Mechanistic Explanation of the pH Dependence and Onset Potentials for Hydrocarbon Products from Electrochemical Reduction of CO on Cu (111). *J. Am. Chem. Soc.* **138**, 483-486 (2016).
11. Li, Z. J., *et al.* Tuning the Spin Density of Cobalt Single-Atom Catalysts for Efficient Oxygen Evolution. *ACS Nano* **15**, 7105-7113 (2021).
12. Mathew, K., Kolluru, V. S. C., Mula, S., Steinmann, S. N.&Hennig, R. G. Implicit self-consistent electrolyte model in plane-wave density-functional theory. *J. Chem. Phys.* **151**, 234101 (2019).
13. Honkala, K., et al. Ammonia synthesis from first-principles calculations. *Science* **307**, 555-558 (2005).
14. Melander, M. M. Grand Canonical Rate Theory for Electrochemical and Electrocatalytic Systems I: General Formulation and Proton-coupled Electron Transfer Reactions. *J. Electrochem. Soc.* **167**, 116518 (2020).
15. He, Z. D., Chen, Y. X., Santos, E. & Schmickler, W. The Pre-exponential Factor in Electrochemistry. *Angew. Chem. Int. Ed.* **57**, 7948-7956 (2018).
