# Supplementary material for: Potential-dependent transition of reaction mechanisms for oxygen evolution on layered double hydroxides
Source: Nat Commun. 2023 Jul 15;14:4228. doi: 10.1038/s41467-023-40011-8 (PMC10349880; doi:10.1038/s41467-023-40011-8)
Supplement: Supplementary file 3 — Description of Additional Supplementary Files [file 41467_2023_40011_MOESM3_ESM.pdf]

Description of Additional Supplementary Files

Supplementary Data 1 Computational Model Data
